# Characterization of phenolic compounds from *Eugenia supra-axillaris* leaf extract using HPLC-PDA-MS/MS and its antioxidant, anti-inflammatory, antipyretic and pain killing activities *in vivo*

**DOI:** 10.1038/s41598-019-46946-7

**Published:** 2019-07-31

**Authors:** Nesrine M. Hegazi, Mansour Sobeh, Samar Rezq, Mohamed A. El-Raey, Malak Dmirieh, Assem M. El-Shazly, Mona F. Mahmoud, Michael Wink

**Affiliations:** 1 Department of Phytochemistry and Plant Systematics, Division of Pharmaceutical Industries, National Research Center, Dokki, Cairo Egypt; 2 Institute of Pharmacy and Molecular Biotechnology, Heidelberg University, Heidelberg, Germany; 3 AgroBioSciences Research Division, Mohammed VI Polytechnic University, Lot 660–Hay Moulay Rachid, 43150 Ben-Guerir, Morocco; 4 Department of Pharmacology and Toxicology, Faculty of Pharmacy, Zagazig University, Zagazig, Egypt; 5 Department of Pharmacognosy, Faculty of Pharmacy, Zagazig University, Zagazig, Egypt

**Keywords:** Drug screening, Pharmaceutics

## Abstract

Reactive oxygen species (ROS) are involved in the pathophysiology of several health disorders, among others inflammation. Polyphenols may modulate ROS related disorders. In this work, thirty-two phenolic compounds were tentatively identified in a leaf extract from *Eugenia supra-axillaris* Spring. ex Mart. using HPLC-MS/MS, five of which were also individually isolated and identified. The extract displayed a substantial *in vitro* antioxidant potential and was capable of decreasing ROS production and hsp-16.2 expression under oxidative stress conditions *in vivo* in the *Caenorhabditis elegans* model. Also, the extract showed higher inhibitory selectivity towards COX-2 than COX-1 *in vitro* with higher selectivity towards COX-2 than that of diclofenac. The extract also exhibited anti-inflammatory properties: It attenuated the edema thickness in a dose dependent fashion in carrageenan-induced hind-paw odema in rats. In addition, the extract reduced the carrageenan-induced leukocyte migration into the peritoneal cavity at the highest dose. Furthermore, the extract showed antipyretic and analgesic activities in a mouse model. *Eugenia supra-axillaris* appears to be a promising candidate in treating inflammation, pain and related oxidative stress diseases.

## Introduction

Reactive oxygen species (ROS) are chemically reactive compounds, among them hydroxyl radicals (^•^OH), superoxide anion radicals (O_2_^•−^), peroxyl radicals (RO_2_^•^) and hydrogen peroxide (H_2_O_2_). Oxidative stress arises when the ROS production is beyond the detoxification capacity of the cells and may prompt lipid peroxidation, damage or modify DNA and proteins. When ROS oxidize the DNA base guanosine to 8-oxoguanosine point mutation may be generated, leading to health conditions, such as cancer. ROS species are also released from activated neutrophils and macrophages and partly contribute to inflammation injury which consequently leads to tissue injury from damaged macromolecules and lipid peroxidation of membranes^1,2^.

Furthermore, ROS stimulate cytokine release which stimulate enrollment of additional neutrophils and macrophages. Therefore, inflammatory processes are mediated and provoked by free radicals. Apparently, natural antioxidants and radical scavengers can reduce inflammation and may help to protect against various types of oxidative damage, which are associated with diseases such as cancer, diabetes, inflammation, liver damage, cardiovascular disorders, and aging^1,2^.

Polyphenols are well-known constituents of the plant kingdom and are the most abundant secondary metabolites of plants, with more than 8000 known phenolic structures^1,2^. They are natural antioxidants with powerful redox properties and thus they act as reducing agents, metal chelating agents, hydrogen donators as well as singlet oxygen quenchers. Because they can form multiple hydrogen and ionic bonds with proteins, they can modulate the biological activity of various proteins. Therefore, polyphenols exhibit a wide array of pharmacological properties, among them hepatoprotective, antidiabetic, and anti-inflammatory activities. These aforementioned activities indicate that polyphenolic compounds can be used to develop new pharmaceuticals^3^.

*Eugenia supra-axillaris* (Myrtaceae) is an evergreen tree, which originates from Brazil. It is cultivated in tropical and subtropical countries. Thirteen phenolic compounds were identified in the wood, among them isoquercetin, rhamnoside 3-sulphate, 3-*O*-monomethoxyellagic acid 4′-*O*-α-rhamnopyranoside-3″-*O*-sulphate, ellagic acid and others^4^. In another study, myricetin, quercetin, and their glycosides, pinocembrin and nilocitin along with gallic, ellagic and 5-*O*-monogalloylquinic acids were characterized in leaf extract and the latter showed appreciable hepatoprotective properties^5^. In addition, chemical and biological activities of the leaf essential oils were also reported^6,7^.

In this study, we characterized the phenolic metabolites from *Eugenia supra-axillaris* leaves using HPLC-MS/MS. Additionally; we evaluated the antioxidant activity of the extract *in vitro* and *in vivo* using the model organism *Caenorhabditis elegans*. In addition, anti-inflammatory, antipyretic, and pain killing activities were investigated in rat and mouse models.

## Materials and Methods

### Plant material

The leaves of *E. supra-axillaris* were collected from El-Zohreya Botanical Garden, Horticulture Institute, Cairo, Egypt in November 2015. The plant was kindly identified by Dr. Mona Marzouk (Associate Professor of Taxonomy at National Research Centre, N.R.C., Egypt). A voucher specimen is kept at the N.R.C. herbarium with the number M111.

### Extraction and fractionation

Fresh leaves (1.5 kg) from *E. supra-axillaris* were homogenized in a MeOH-H_2_O (3:1) mixture (3 × 4 L). The obtained extract was filtered and dried under reduced pressure to give a yield of 120 g dried extract. The obtained extract was re-dissolved in water (500 mL) and defatted with *n*-hexane (3 × 500 mL). The obtained defatted extract was collected, dried under reduced pressure and frozen at –70 °C and then lyophilized yielding 90 g of a fine dried powder. The defatted extract was also investigated for its phenolic content by two-dimensional paper chromatography (TDPC) using *n*-butanol: acetic acid: water, 4:1:5 upper layer (BAW) as the first developing solvent and 6% acetic acid as the second solvent.

### HPLC-MS/MS

A Thermofinnigan (Thermo Electron Corporation, USA) coupled with an LCQ-Duo ion trap mass spectrometer with an ESI source (ThermoQuest) system was utilized. A C18 reversed-phase column (Zorbax Eclipse XDB-C18, rapid resolution, 4.6 × 150 mm, 3.5 µm, Agilent, USA) was used to separate the analytes. A gradient of water and acetonitrile (ACN) (0.1% formic acid each) was applied from 5% to 30% ACN over 60 min with flow rate of 1 mL/min with a 1:1 split before the ESI source. The samples were injected automatically using autosampler surveyor ThermoQuest. The instrument was controlled by Xcalibur software (Xcalibur^TM^ 2.0.7, Thermo Scientific). The MS operated in the negative mode as before^8^. The ions were detected in a full scan mode and mass range of 50–2000.

### Isolation and identification of the phenolic metabolites

The defatted aqueous methanol extract of the leaves (20 gm) was dissolved in water and applied to a polyamide 6 s column and eluted with H_2_O followed by H_2_O-MeOH mixtures of decreasing polarities. The collected fractions were investigated by TDPC using BAW as the first solvent and 6% acetic acid as the second solvent. Compound **1** (Table 1) was isolated from fraction I by crystallisation. Compounds **7** and **9** (Table 1) were isolated from fraction II by preparative paper chromatography using BAW as an eluent. Similarly, compounds **16** and **23** (Table 1) were separated from fraction III and IV, respectively. The isolates were identified according to their UV spectral data, ^1^H- and ^13^C-NMR assignments.

**Table 1:** Tentative identification of phenolic compounds in *E. supra-axillaris* leaves by HPLC-ESI-MS/MS.

No.	R_t_	[M-H]^−^	MS/MS	Proposed structure
1	1.93	169	125	Gallic acid*
2	2.06	331	169	Galloyl glucose
3	2.10	331	169	Galloyl glucose
4	2.4	353	191, 179	5-*O*-Caffeoylquinic acid
5	3.86	353	191, 179	3-*O*-Caffeoylquinic acid
6	4.21	353	191, 179	4-*O*-Caffeoylquinic acid
7	4.32	483	331, 169	2, 6 di-*O*-(α/β)-^4^*C*_1_- galloyl glucose*
8	4.57	337	191, 163	*p*-Coumaroylquinic acid
9	4.75	483	331, 169	2,3 di-*O*-(α/β)-^4^*C*_1_- galloyl glucose*
10	5.06	757	301, 613, 633	Galloyl decarboxy valoneoyl glucoside
11	9.94	183	183, 169	Methyl gallate
12	11.07	563	417, 285	Kaempferol pentosyl rhamnoside
13	15.87	385	223, 178	Sinapic acid hexoside
14	16.44	399	273, 179	Monolactonoside didecarboxylated valoneoic acid
15	17.97	275	275, 257, 229	3,4,8,9,10-pentahydroxy-6-oxobenzo[c]chromene
16	17.24	595	463	Myricetin-3-*O*-xylopyranosyl(1 → 2)-*α*-^1^C_4_-rhamnopyranoside*
17	21.89	479	317	Myricetin hexoside I
18	22.84	367	191, 179	Feruloylquinic acid
19	23.03	479	317	Myricetin hexoside II
20	23.56	449	317	Myricetin pentoside I
21	24.54	449	317	Myricetin pentoside II
22	24.81	497	169, 183, 313, 341	Gallic acid methyl ester (-*O*-galloyl) hexoside
23	26.42	463	317	Myricetin 3-*O*-*α*-^1^C_4_-rhamnopyranoside*
24	27.71	463	317	Myricetin rhamnoside I
25	28.8	463	317	Myricetin rhamnoside II
26	31.80	491	359	Tri-*O*-methyl myricetin pentoside
27	32.78	447	301	Quercetin deoxyhexoside
28	38.33	431	285	Kaempferol deoxyhexoside
29	40.33	615	179, 301, 463	Myricetin galloyl-rhamnoside I
30	43.13	615	179, 301, 463	Myricetin galloyl-rhamnoside II
31	49.2	593	447, 285	Kaempferol coumaroyl-hexoside
32	51.98	657	495, 329	Caffeoyl digalloyl quinic acid

*Compounds isolated and identified during this study.

### *In vitro* antioxidant evaluation

Determination of total phenolic contents, DPPH radical scavenging activity and FRAP ferric reducing antioxidant power assays were done as described^9^. The total phenolic content was estimated using Folin-Ciocalteu method. In brief, 20 µL solution of the plant extract was mixed with 100 µL Folin-Ciocalteu reagent. At ambient temperature, the mixture was incubated for 5 min and then 80 µL of 7.5% Na_2_CO_3_ solution was added. The mixture was then left for 30 min in the dark and the absorbance was monitored using a microplate reader (Biochrom Asys UVM 340) at 735 nm. The results were presented as gallic acid equivalent (GAE) in mg/g of extract. Total antioxidant capacity (TAC) assay was determined using a commercially available TAC ELISA kit (MBS726896, my biosource, Inc., San Diego, CA, USA) according to the manufacturer’s instructions with ascorbic acid as the reference standard to estimate TAC as described^10^.

### *In vivo* antioxidant activities (*Caenorhabditis elegans* experiments)

The worms were maintained under standard conditions as described before^11^. In brief, age synchronized cultures were obtained by sodium hypochlorite treatment of gravid adults; the eggs were kept in M9 buffer for hatching. The obtained larvae were then transferred to S-media containing *E. coli* OP50 (D.O_600_ = 1.0)^12^. We here used Wild type (N2), TJ375 [P*hsp-16.2: GFP(gpls1)*] and TJ356 strains to perform the experiments as described before^11^. The worms were provided by the *Caenorhabditis* Genetic Centre (CGC), University of Minnesota, U.S.A. In brief, Age synchronized worms (TJ356, L1 stage, grown in S-medium) were used to quantify the subcellular DAF-16: GFP localization. The worms were treated with three different doses (50, 100 and 200 µg/mL) at 20 °C for 24 h in S-medium. Fluorescence microscopy was used to collect the images. The worms were classified to cytosolic, intermediate, and nuclear based on the localization of the fusion DAF-16::GFP.

### Anti-inflammatory experiments

#### *In vitro* anti-inflammatory experiments

The lipoxygenase (LOX) suppression potential was analysed according to Abdelall *et al*.^13^ using a lipoxygenase inhibitor screening assay kit (Cayman Chemical, AnnArbor, MI, USA). Cyclooxygenases (COX-1 and COX-2) were measured by an enzyme immunoassay (EIA) kit (Cayman Chemical, AnnArbor, MI, USA) according to the manufacturer’s instruction. COX-2 selectivity index (SI values) which is defined as IC_50_ (COX-1)/IC_50_ (COX-2) was estimated and compared to the reference compounds celecoxib, indomethacin and diclofenac as previously described^10^.

#### *In vivo* anti-inflammatory experiments

Adult male Wistar rats (140–160 g) and Swiss albino mice (20–25 g) obtained from the Faculty of Veterinary Medicine, Zagazig University, Egypt were used in this study. They were housed under constant experimental conditions of humidity (55%), temperature (23 °C), and 12 h light/dark cycle. Animals were left for one week for acclimatization before performing the experiments and were supplied with water and a commercially available regular chow diet *ad libitum*. The study protocol was approved by the Ethical Committee of the Faculty of Pharmacy, Zagazig University for Animal Use (P 9-12-2017) and performed according to the guidelines of the US National Institutes of Health on animal care and use.

#### Carrageenan-induced hind-paw odema

The vehicle (10 mL/kg), *E. supra-axillaris* aqueous methanol extract (200 mg/kg and 400 mg/kg, p.o.) or diclofenac (10 mg/kg) were orally gavaged to rats (n = 6/group) 1 h before the induction of inflammation. Afterwards, hind paw odema was induced in the right leg of the rats by injecting freshly prepared carrageenan solution (1% in 0.9% NaCl, 0.1 mL), into the sub plantar tissue. The thickness (mm) of the right leg hind paw was measured in the dorsal- plantar axis by a calliper ruler before and after the carrageenan injection at hourly intervals for 6 h and then at 24 h. The cumulative anti-inflammatory effect during the whole experiment period (0–24 h) was evaluated by detecting the area under the curve of changes in paw thickness-time curve (AUC_0–24_).

#### Migration of leukocyte to peritoneal cavity in mice

This test was carried out according to the method of Silva-Comar *et al*.^14^. Swiss albino mice (n = 5–8/group), was assigned into four groups and orally given the vehicle (1 mL/100 g, p.o.) or extract (200 mg/kg and 400 mg/kg, p.o.). Thirty min later, the animals were injected intraperitoneally with 0.1 mL carrageenan solution (500 *μ*g/mice) or 0.1 mL sterile saline. Diclofenac (10 mg/kg, p.o.) was used as an anti-inflammatory reference compound. After 3 h, the animals were euthanized, and the peritoneal cavity was washed with 3 mL of phosphate-buffer saline (PBS) containing 1 mM ethylenediamine tetra-acetic acid (EDTA). The number of leukocytes in the peritoneal cavity wash was determined using a haemocytometer and expressed as number of cells/mL.

### Analgesic activity experiments

#### Peripheral anti-nociceptive activity experiment

Acetic acid writhing test in mice was used to evaluate the peripheral analgesic activity of the extract according to the method of Nakamura *et al*.^15^. Briefly, mice were assigned into 4 groups (5–7 mice). Group (1) was pre-treated with the vehicle (1% Tween 80, 10 mL/kg) and served as negative control. Groups (2) and (3) pre-treated with the extract (200 and 400 mg/kg, p.o. respectively) and group (4) pre-treated with diclofenac (10 mg/kg, a reference drug) 1 h prior i.p. injection of 0.7% acetic acid (1 mL/100 g). The total number of writhes was recorded for 25 min.

#### Central anti-nociceptive activity (Hot plate test)

The possible central analgesic activity of the extract was tested using hot plate method^16,17^. Four groups of mice (6 each) were used in the present experiment and received either the extract (100 or 200 mg/kg, p.o.) or the vehicle (10 mL/kg, p.o.). The opioid analgesic nalbuphine was given to another group of mice as reference central analgesic. Each mouse was put on a hot plate heated at 55 ± 1 °C, 60 min after giving the drugs or vehicle. Mice were observed for their response to heat of the hot plate (lapping of the fore and hind paws, hind paw rising or jumping). The required time for the animals to show the first response to the heat was recorded, at baseline and at 1, 2, 3, and 4 h following the administration of vehicle or drugs.

#### Acetic acid-induced mouse vascular permeability

The acetic acid-induced mouse vascular permeability was carried out as previously described^18^. Mice were assigned into four groups (6 mice/each group). They were pre-treated with the extract in two dose levels (200 mg/kg, and 400 mg/kg, p.o.), diclofenac (20 mg/kg) or vehicle. After 1 h of pretreatment, 0.2 mL Evans blue (0.25% solution in normal saline) was injected in the tail vein. Thirty min later, acetic acid (0.6% in normal saline, 1 mL/100 g) was injected in the peritoneal cavity of mice. Another group of mice was injected with normal saline only and served as normal control. After another 30 min, the mice were euthanized by cervical dislocation. The peritoneal cavity of each mouse was washed with 3 mL saline and the washing was then centrifuged at 3000 *g* for 10 min. Evans blue dye content of the supernatant which is proportional to the vascular permeability was detected at 610 nm using a plate reader (Biotech, Vt, USA).

#### Induction of pyrexia in mice

In this experiment 4 groups of mice were used (n = 6). Pyrexia was induced in mice as described previously with modifications^19,20^. The initial body temperature of the rectum was recorded for each mouse using a lubricated digital thermometer. Next, Brewer’s yeast suspension was prepared in normal saline (30%) and then mice were injected with yeast suspension (1 mL/100 g) subcutaneously behind the neck. 18 h later rectal temperature was recorded again (T_0_) and mice showed a higher record by at least 0.5 °C were included in the study. Pyretic mice were then given the extract (200 mg/kg, and 400 mg/kg, p.o.), paracetamol (150 mg/kg) or vehicle. The rectal temperature was reordered again at 30 min, 1, 2, 3 and 24 h post-treatment.

#### Statistical analysis

The data of the present study are expressed as mean ± SEM. Results of the experiments were statistically analysed using GraphPad Prism software, version 5.00 (GraphPad Software, Inc. La Jolla, CA, USA). The statistical difference among different groups was evaluated using Analysis of Variance (ANOVA) or repeated-measures analysis of variance (RM-ANOVA) followed by Tukey’*s post hoc* test and Student’s *t*-test. The considered level of significance was *p* < 0.05.

## Results

### Phenolic profile of *E. supra-axillaris* extract by LC-MS/MS

**Figure 1:**
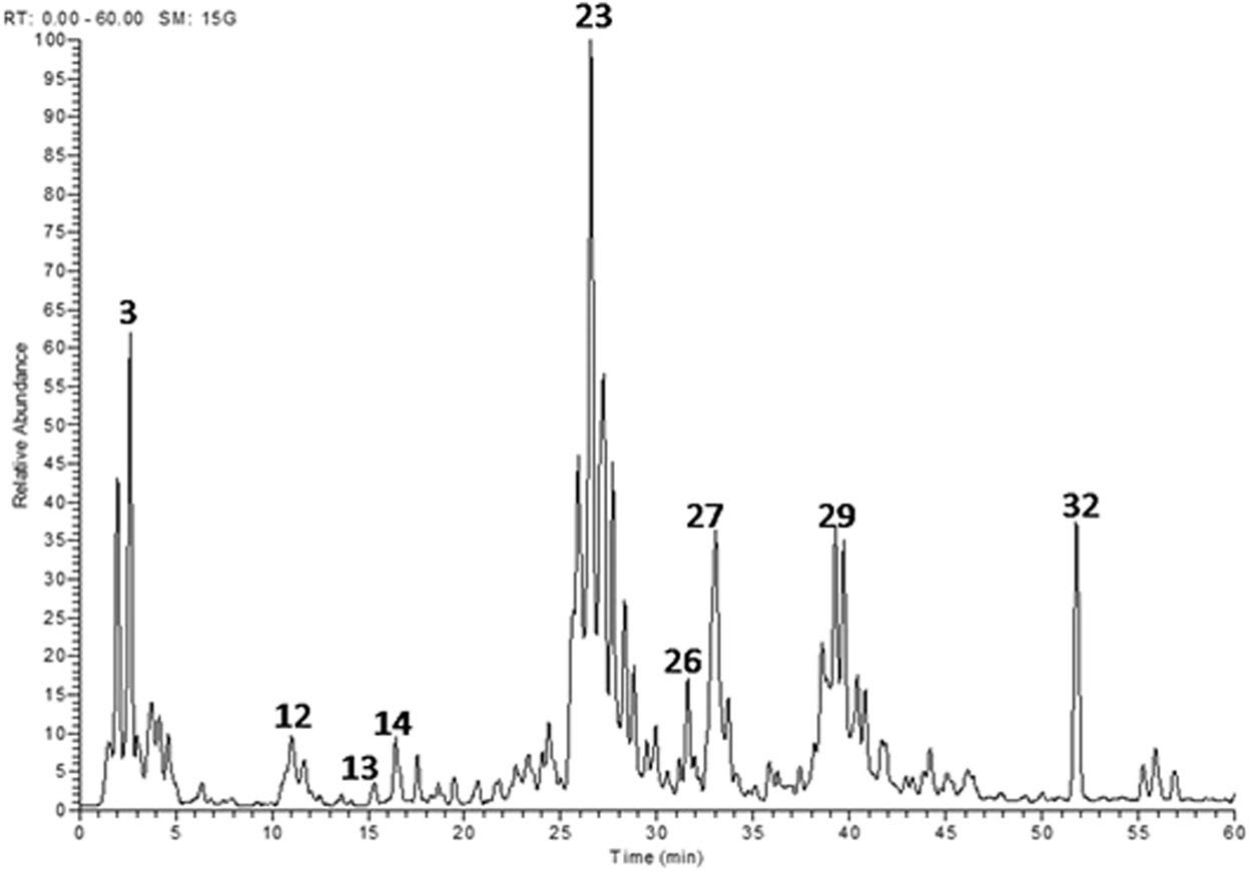
LC-MS/MS profile of a water/methanol extract from *E. supra-axillaris* leaves. Numbering of peaks as in Table 1.

**Figure 2:**
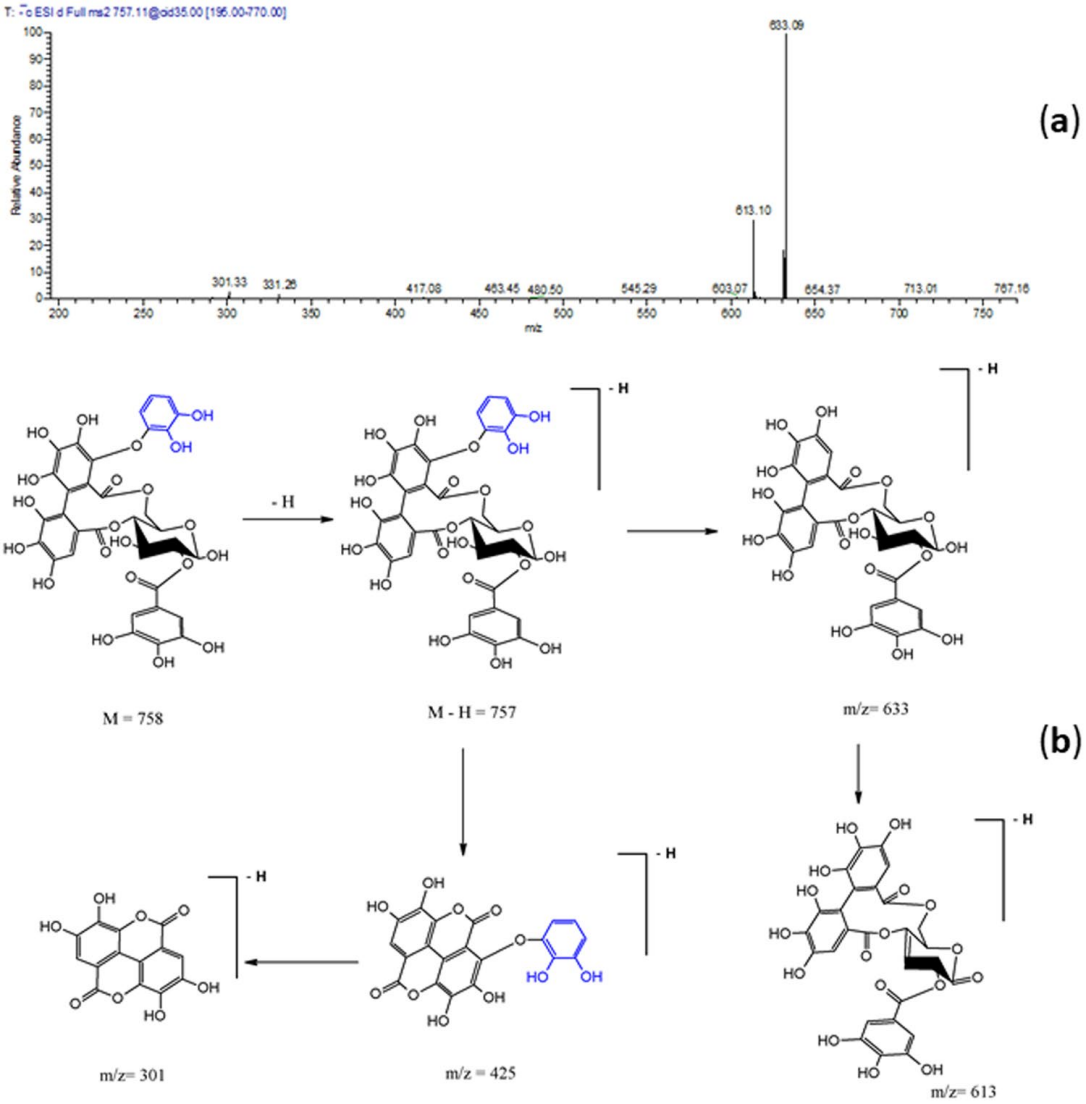
(**a**) Negative ion ESI-MS/MS spectra of galloyl decarboxy valoneoyl glucoside at [M-H]^−^ *m*/*z* 757. (**b**) A putative fragmentation pattern is illustrated.

**Figure 3:**
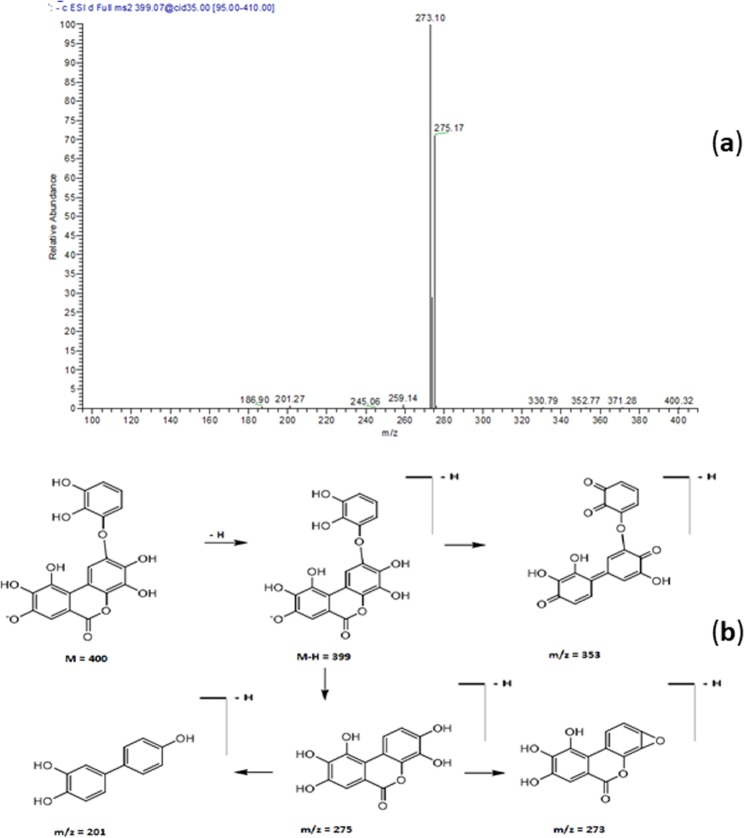
(**a**) ESI-MS/MS spectra in the negative mode of monolactonoside didecarboxylated valoneoic acid at [M-H]^−^ *m*/*z* 399. (**b**) A putative fragmentation pattern is shown.

Profiling of the polyphenolic metabolites in *E. supra-axillaris* leaf extract was performed by HPLC-ESI-MS/MS. In total, thirty-two secondary metabolites were characterized and tentatively identified based on their retention times, molecular weight, and fragmentation pattern as well as comparison with reported data (Fig. 1 and Table 1**)**. A molecular ion peak at retention time 5.06 min showed an [M-H]^−^ *m/z* 757 and daughter ions at *m/z* 301, 613 and 633 was tentatively assigned to galloyl decarboxyvaloneoyl glucoside (Table 1 and Fig. 2). Compound **14** exhibited a [M-H]^−^ at *m/z* 399 and a main daughter ion at *m/z* 275; it was tentatively characterized as monolactonoside didecarboxylated valoneoic acid **(**Fig. 3**)**.

### Isolation and structure elucidation

Five phenolic compounds were isolated and identified based on their chromatographic behavior, UV spectral, ^1^H-and ^13^C- NMR data which were consistent with those previously reported for gallic acid (**1)**, myricetin-3-*O*-*β*-^4^C_1_-xylopyranosyl (1 → 2)-*α*- ^1^C_4_-rhamnopyranoside (**16)**, myricetin -3-*O*-*α*-^1^C_4_-rhamnopyranoside (**23)**, 2,3 di-*O*-(α/β)-^4^*C*_1_- galloyl glucose, nilocitin (**7)**, and 2, 6 di-*O*-(α/β)-^4^*C*_1_- galloyl glucose (**9)**^5,21^.

### Antioxidant activities

The extract showed substantial antioxidant activities *in vitro* in DPPH and FRAP assays when compared to positive controls ascorbic acid and quercetin, respectively (Table 2). The total phenolic content amounted 335 mg GAE/g extract according to the Folin-Ciocalteu method. Additionally, the total antioxidant capacity (TAC) assay of the extract was 1.5-fold higher than that of the solid antioxidant compound, ascorbic acid (Table 2**)**.

**Table 2:** Antioxidant activities of *E. supra-axillaris* leaf extract.

Sample	DPPH	FRAP	TAC
	(EC_50_ µg/mL)	(mM FeSO_4_ equivalent/mg extract)	U/L
Extract	9.17 ± 0.88	17.12 ± 0.43	41.25
Ascorbic acid	3.23 ± 0.75	—	28.12
Quercetin	—	21.08 ± 0.93	—

**Figure 4:**
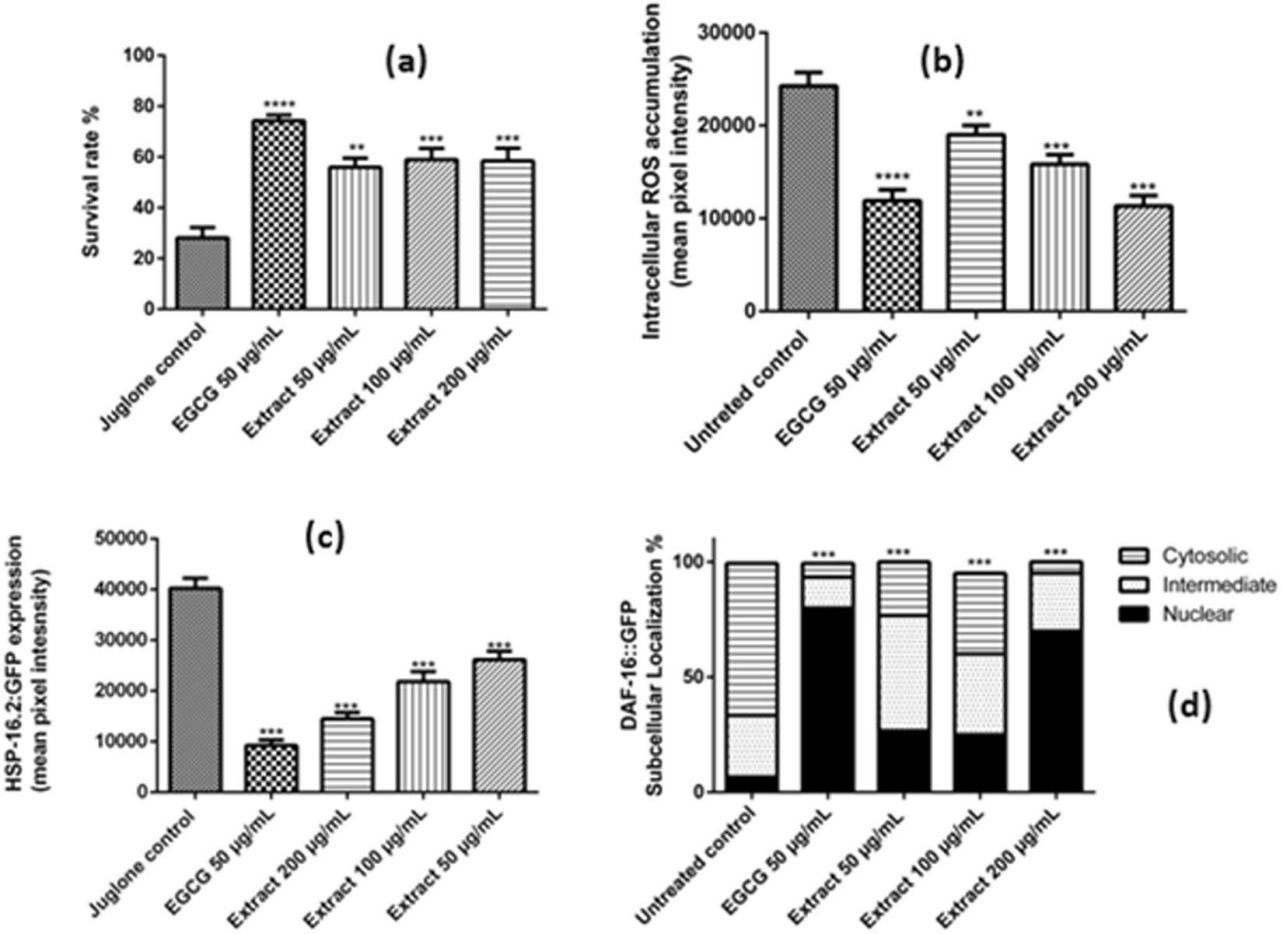
*In vivo* antioxidant activities of *E. supra-axillaris* in *C. elegans*. (**a**) Survival rate of wild type N2 worms under a lethal dose of juglone (80 μM). The results are expressed as percentage of survival (mean ± SEM, n = 3; untreated worms have a survival of 100%). **p < 0.01, ***p < 0.001, ****p < 0.0001 related to control was analysed by one-way ANOVA. (**b**) Intracellular ROS accumulation in N2 strains using H2DCF-DA as an indicator. Data are presented as the percentage of fluorescent pixels related to control (n = 3). The extract reduced significantly the ROS content in a dose dependant manner. (**c**) *Phsp*-16.2::GFP expression in the mutant strains TJ375. The extract reduced the levels of *Phsp*-16.2:GFP significantly in a dose dependant manner. Data are presented as the mean of fluorescent pixels related to control. (**d**) DAF-16::GFP translocation in the mutant strains TJ35. DAF-16 subcellular localization pattern is shown in terms of percentage of worms exhibiting a cytosolic, intermediate, and nuclear localisation. ***p < 0.001 related to control was analysed by one-way ANOVA.

To examine the protective effects of the extract *in vivo*, we monitored the survival rate of the wild type *C. elegans* worms N2 under a lethal dose of 80 µM of the pro-oxidant juglone. Worms which were pretreated with the extract had enhanced survival rates when compared to juglone group, which was treated with juglone alone. Epigallocatechin gallate (EGCG) was used as positive control (Fig. 4a**)**. We also investigated the influence of the extract on the intracellular accumulation of ROS. *E. supra-axillaris* extract was able to decrease the levels of ROS *in vivo* in a dose dependent manner (Fig. 4b**)**.

To further investigate the antioxidant activity *in vivo* of the extract, we used a sensitive oxidative stress sensor *hsp-*16.2. Under oxidative or heat stress, *hsp-16.2* acts as chaperon to recognize and degrade unfolded proteins. After exposing the nematodes to 20 μM juglone for 24 h, *hsp-16.2* is highly expressed. The extract was able to reduce *hsp-16.2* expression in a concentration-dependant manner (Fig. 4c**)**. Thus, the extract can alleviate oxidative stress in worms and its compounds show bioavailability.

DAF-16 (a member of the FOXO transcription factor group) is crucial in several signaling pathways that regulate the stress response, age-related diseases as well as other important biological processes. To get an insight in the molecular mode of action of the tested extract, the transgenic *C. elegans* strain TJ356 was used to investigate the influence of the extract on the subcellular localization of DAF-16, which is in the cytosol when inactive. The extract caused a translocation of FOXO transcription factor DAF-16 from the cytoplasm to the nucleus indicating that the *in vivo* antioxidant capacity of the extract may involve the DAF-16/FOXO regulated signalling pathway (Fig. 4d**)**.

### Anti-inflammatory effects

#### *In vitro* anti-inflammatory activities

Cyclooxygenase (COX) is a key enzyme in the biosynthesis of prostaglandin from arachidonic acid. COX has two main isoforms: COX-1 and COX-2. Inflammation mediated pathologies are related to COX-2 over-expression. The *E. supra-axillaris* extract suppressed both COX-1 and COX-2 *in vitro* with greater selectivity towards COX-2 than COX-1. Interestingly, the extract has a much more COX-2 selectivity than that of diclofenac (Table 3**)**. Moreover, the extract has nearly double the potency of zileuton, the reference LOX inhibitor to inhibit lipoxygenase enzyme *in vitro* (Table 3).

**Table 3:** The *in vitro* effects of *E. supra-axillaris* extract on COX-1, COX-2 and 5-LOX.

Assay	5-LOX	COX-1	COX-2	SI
	IC_50_ (µg/mL)			
Extract	1.61	4.11	0.065	63.2
Celecoxib	—	17.5	0.046	380.4
Diclofenac	2.11	3.8	0.7	5.42
Indomethacin	—	0.034	0.45	0.075
Zileuton	3.21	—	—	—

SI is COX selectivity index which is defined as IC_50_ (COX-1)/IC_50_ (COX-2).

#### Inflammation: Effects of the extract on carrageenan-induced paw edema in rats

**Figure 5:**
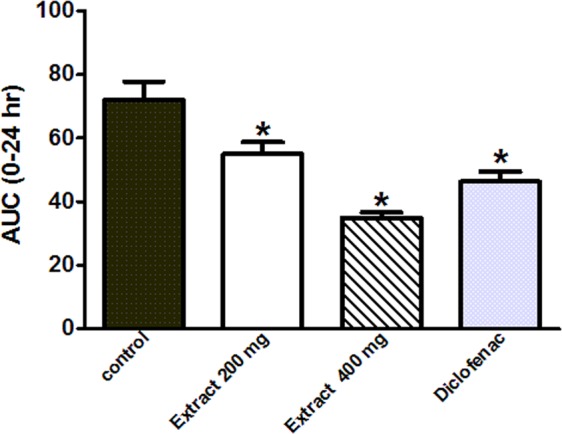
Effect of *E. supra-axillaris* leaf extract (200 and 400 mg/kg, p.o.) or diclofenac (10 mg/kg, p.o) on carrageenan induced-hind paw edema in rats. Data represents the AUC_0–24_ and is expressed as mean ± S.E.M (n = 5). *p < 0.05 vs. control values using Analysis of Variance (ANOVA) followed by Tukey *post hoc* test.

Animals, injected with 0.1 mL carrageenan (1% in 0.9%, sub-planter), showed increased paw thickness when measured each hour for 5 h and at 24 h after injection. The highest increase was observed 4 h post injection to reach 3.9 ± 0. 28 mm over base line readings (paw thickness measured before carrageenan injection). Rats pretreated 1 h earlier with the extract (200 and 400 mg/kg, p.o.) showed a significant decrease in edema thickness in a dose-dependent fashion with more potent activities than the standard anti-inflammatory drug, diclofenac (Fig. 5**)**.

#### Effects of *E. supra-axillaris* extract on mouse carrageenan-induced leukocyte migration

**Figure 6:**
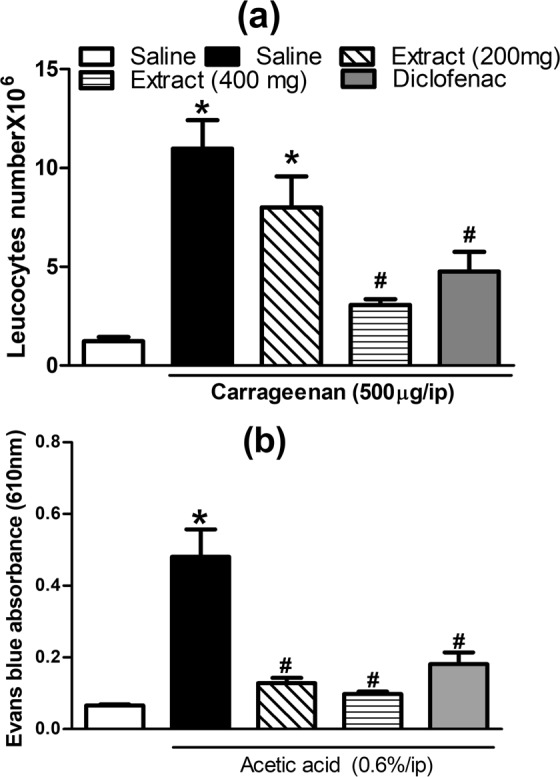
Effect of carrageenan (500 µg, i.p.) on leukocyte mobilization into the peritoneal cavity of mice (total number × 10^6^) with or without 1 h prior treatment with the extract (200 and 400 mg/kg, p.o.) or diclofenac (10 mg/kg, p.o). Each value represents mean ± S.E.M (n = 5–7). *p < 0.01 vs. vehicle (saline) values, ^#^*p* < 0.01 vs control (carrageenan treated group) using Analysis of Variance (ANOVA) followed by Tukey *post hoc* test.

Pretreatment with the extract attenuated carrageenan (500 μg/cavity, i.p., 0.1 mL) induced leukocyte recruitment into the peritoneal cavity in mice. Noteworthy, the response to the extract was more potent than that obtained in animals treated with diclofenac, the reference drug, 1 h prior to carrageenan challenge (Fig. 6**)**.

#### Effects of *Eugenia supra-auxillaris* on acetic acid-induced vascular permeability in mice

As shown in Fig. 6, acetic acid injection in mice (0.6%, ip) increased vascular permeability represented as significantly (P < 0.001) higher Evans blue absorbance in the peritoneal cavity exudate compared to saline injected mice (0.48 ± 0.08 vs 0.07 ± 0.003). This effect was attenuated in mice pretreated with *E. supra-auxillaris* extract (200 and 400 mg/kg, p.o) 1h r prior acetic acid injection by 73 and 80%, respectively. Additionally, the reference standard, diclofenac sodium achieved 63% lower reading compared to control mice (Fig. 6).

#### Pain-killing properties: Effects of the extract on acetic acid-induced writhing response in mice

**Figure 7:**
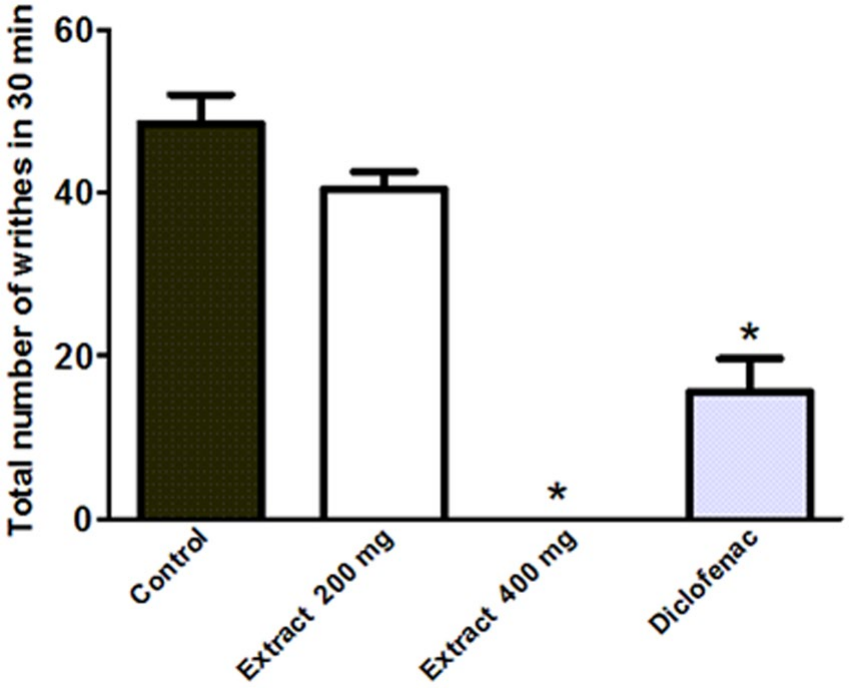
Effects of the extracts (100 and 200 mg/kg, p.o.) or diclofenac (10 mg/kg, p.o.) on the writhing response to acetic acid (0.7%, 1 mL/100 g) in mice. Results are expressed as mean ± S.E.M (n = 5–8). *p < 0.001 vs. control values using Analysis of Variance (ANOVA) followed by Tukey *post hoc* test.

Oral pretreatment with the extract revealed a weak potency at the lower dose (200 mg/kg, p.o) but completely abolished acetic acid (0.7% acetic acid, 1 mL/100 g) induced writhes in mice when used at a higher dose (400 mg/kg) to achieve zero writhes in the overall 30 min observation period. Additionally, mice pretreated with diclofenac (10 mg/kg, p.o.), the reference standard, showed 67% reduction of the control writhes (Fig. 7**)**.

#### Analgesic properties: Effects of the extract on hot plate test in mice

**Figure 8:**
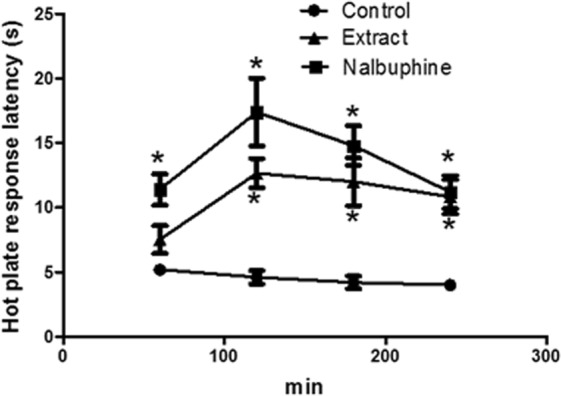
Effect of vehicle, *E*. *supra-axillaris* extract (200 mg/kg, p.o.) or nalbuphine (10 mg/kg, p.o.) administration on hot plate response latency (s) measured 1–4 h in mice. Each value represent mean ± S.E.M (n = 5–8). *p < 0.001 vs. control values using Analysis of Variance (ANOVA) followed by Tukey *post hoc* test.

Mice pretreated with the extract (200 mg/kg, p.o.) and the reference standard nalbuphine (10 mg/kg, i.p.), showed a longer response latency when measured at 1, 2, 3 and 4 h after administration to reach its peak at the 2 h time point (2.8 and 3.8 fold of control., respectively). While the effect was significant (*p* < 0.001) at all-time points for nalbuphine, the effect of extract was only significant 2, 3 and 4 h post treatment (Fig. 8**)**.

#### Antipyretic activities: Effect of the extract on Brewer’s yeast induced pyrexia in mice

Brewer’s yeast injection in mice raised rectal body temperature of injected mice when measured 18 h after injection. Mice pretreated with *E. supra-axillaris* extract (200 and 400 mg/kg, p.o.) showed significantly lower rectal temperatures starting from 1 h post treatment (*p* < 0.001). This antipyretic effect was similar in the two studied doses and comparable to the effect obtained in mice treated with paracetamol (150 mg/kg), the antipyretic standard drug (Table 4**)**.

**Table 4:** Effect of *E. supra-axillaris* extract on Brewer’s yeast induced pyrexia in mice.

Sample	Rectal temperature 18 h after yeast injection	Rectal temperature recorded following different treatments				
		3 min	1 h	2 h	3 h	24 h
Control	38.2 ± 0.24	38.4 ± 0.13	38.4 ± 0.12	39 ± 0.23	38.8 ± 0.31	38.2 ± 0.16
Extract (200 mg/kg)	38.2 ± 0.27	37.8 ± 0.26	37.1 ± 0.35*	37 ± 0.27*	37 ± 0.25*	35.8 ± 0.1*
Extract (400 mg/kg)	38.5 ± 0.34	38 ± 0.38	36.9 ± 0.59*	36.9 ± 0.4*	37 ± 0.38*	36.3 ± 0.19*
Paracetamol (150 mg/kg)	38.5 ± 0.22	38.1 ± 0.2	37.5 ± 0.3	37 ± 0.3*	37 ± 0.24*	36.3 ± 0.24*

Each value represents the mean ± S.E.M (n = 5), *p < 0.001 vs. control valuesusing Analysis of Variance (ANOVA) followed by Tukey *post hoc* test.

## Discussion

In the current study, a leaf extract from *E. supra-axillaris* exhibited a plethora of pharmacological activities. It scavenged the free radicals *in vitro* in DPPH assay, reduced FeSO_4_ in FRAP assay and exhibited total antioxidant capacity (TAC) which was 1.5-fold higher than that of the gold antioxidant standard, ascorbic acid.

It also significantly increased the survival rate against the deleterious effects of juglone, reduced the intracellular ROS and juglone induced *Phsp*-16.2:GFP levels in a dose dependant manner and induced nuclear translocation of DAF-16::GFP in *C. elegans*. Similar effects have been reported for other plant extracts rich in polyphenols^11^.

Additionally, *E. supra-axillaris* extract exhibited substantial anti-inflammatory activities *in vitro* and inhibited 5-LOX, COX-1, and COX-2 with higher activities than the reference drug compounds. Furthermore, the extract reduced edema thickness, inhibited leukocyte migration, diminished the acetic acid induced writhes and lowered the elevated rectal temperatures in mice. These activities might be attributed to its secondary metabolites, namely myricetin-3-*O*-xylopyranosyl (1 → 2)-*α*-^1^C_4_-rhamnopyranoside, myricetin hexoside, myricetin pentoside, myricetin 3-*O*-rhamnoside, kaempferol deoxyhexoside and others. These polyphenols can interact with various proteins by forming hydrogen and ionic bonds which would explain the pleiotropic effects^1,2,22–27^.

Carrageenan induced edema is a common experimental model used to assess the anti-inflammatory effects of natural products. The extract exerted potent inflammatory effect in a dose-dependent fashion with more potent activities than the standard anti-inflammatory drug, diclofenac which may be attributed to the observed COX-2 inhibition. Similar findings had been reported for *Eugenia brasiliensis*^23^.

We further investigated the anti-inflammatory effect of the extract on carrageenan-induced peritonitis model. In this model, carrageenan, causes the leukocytes recruitment to the peritoneal cavity. Leukocyte recruitment to the inflammation site plays an important role in the development of an inflammatory process. It is a highly regulated process and represents a potential therapeutic target^28^. This study showed that the extract was more potent than diclofenac in reducing the number of inflammatory cells at the inflammation site. Similar findings had been reported for *Eugenia jambolana*^24^.

The released inflammatory mediators during different phases of inflammation stimulate both peripheral and central nociceptive pathways resulting in pain sensation. Tissue injury leads to activation of both COX and LOX and subsequent formation of prostanoids, cytotoxins as well as leukotrienes that will act in both the development of the inflammatory process and the hypernociceptive signal^29^. Surprisingly, the higher dose of the extract completely abolished acetic acid induced writhes in mice to achieve zero writhes in the overall 30 min observation period while mice pretreated with diclofenac, the reference standard, showed 67% reduction of the control writhes. These results may be attributed to the inhibition of COX and the inhibition of the generation of pain mediators mainly, prostaglandins. Similar findings had been reported for *Eugenia jambolana*^24^.

The extract not only exerted a peripheral anti-nociceptive effect but also exerted a central anti-nociceptive effect. The hot-plate test is used in the present study to evaluate the central anti-nociceptive effects. The extract showed longer latency in the response of mice to hot plate, which was significant 2, 3 and 4 h post treatment. However, it is less potent than the reference standard nalbuphine.

Brewer’s yeast induced pathogenic fever by increasing the synthesis of prostaglandin^30^, in the hypothalamus and is utilized in this study to investigate antipyretic effect of the extract. The *extract* in both studied dose levels showed significantly lower rectal temperatures starting from 1 h post treatment and their effects is like that of paracetamol. Therefore, it can be postulated that the extract may interfere with the release of prostaglandin (PGE2) and pyrogenic cytokines^31^. This study showed that the extract inhibits both COX-1 and COX-2 enzymes which lead to inhibition of synthesis of prostaglandins (PGE2). The antipyretic activity of the extract may be attributed to the phenolic components of the extract. Noteworthy, the current results are in a good agreement with those reported from other *Eugenia* species, namely *E. jambolana*, *E. brasiliensis*, *E. uniflora* and other Myrtaceae species *Syzygium aqueum*, *S. jambos* and *S. samarangense*^10,23–27,32^.

## Conclusions

Profiling of the phenolic secondary metabolites of *E. supra-axillaris* leaves was performed utilizing HPLC-MS/MS and led to the dereplication of thirty-two compounds, from which seventeen were reported for the first time in this species. Moreover, five phenolics were individually isolated and identified. The extract exhibited substantial antioxidant, anti-inflammatory, antipyretic and analgesic activities *in vivo*. In conclusion, the witnessed *in vitro* and *in vivo* activities suggest that *E. supra-axillaris* could exert protective properties against oxidative and free radical damage associated with diverse pathological disorders. Further studies are needed, to explore the corresponding mechanisms.
